# The impact of COVID-19 on electronic repeat dispensing (eRD) in general practice

**DOI:** 10.1186/s40545-023-00566-9

**Published:** 2023-05-17

**Authors:** Raman Sharma, Farideh A. Javid

**Affiliations:** 1 Parkside Medical Practice, Horton Park Health Centre, Bradford, BD7 3EG UK; 2 Department of Pharmacy, School of Applied Sciences, University of Huddersfield, Huddersfield, HD1 3DH UK

**Keywords:** COVID-19, General practice, Electronic repeat prescriptions, Electronic repeat dispensing, Repeat prescribing

## Abstract

**Background:**

Electronic repeat dispensing (eRD) has been part of the community pharmacy contact since 2005 and a requirement in the General Medical Services contract since 2019. NHS England highlights benefits of eRD as increased efficiency in general practice of 2.7 million hours annually if 80% of all repeat prescriptions are issued as eRD. Despite clear benefits to patients, community pharmacies and general practices, the uptake of eRD remains low and variable across general practices in West Yorkshire, UK.

**Objectives:**

To investigate the impact of COVID-19 on eRD in general practice and understand the key enablers to its uptake.

**Methods:**

A 19-item questionnaire was developed and piloted during cognitive interviews. A cross-sectional survey was conducted via emails to general practices in West Yorkshire, UK, between July 2020 and November 2020.

**Results:**

Sixty-seven complete responses were received (23 pharmacists, 21 practice managers, 11 general practitioners, seven pharmacy technicians, four advanced practitioners, one prescription clerk). 59% of respondents were aware of eRD uptake in their surgery (mean value 4.56% ± 0.229%). Higher uptake of eRD was demonstrated where the general practice integrated eRD into routine workflows during the repeat prescription reauthorisation process (*P* < 0.001) and where an eRD service lead is nominated (*P* = 0.04).

**Conclusion:**

Utilising eRD in the respective practices should be considered due to potential efficiency gains and the increase in average eRD utilisation observed in the study participating general practices was from 7.2% average uptake in March 2020 to 10.4% November 2020, as the response to COVID-19. The stated benefits of eRD by NHS England of 2.7 million hours per annum predates the roll out of electronic transmission of prescriptions suggesting further research is needed to quantify the efficiency gains in present NHS general practice environments.

## Background

The COVID-19 pandemic had an impact on how general practices were to operate during the lockdown as face-to-face contact was no longer an option and this meant that general practices were to provide healthcare services via an alternative route and moved to the total triage system of healthcare provision. Prioritising workloads to deal with the expected surge in demand for healthcare services was advised by NHS England, and telephone or virtual and video-based triage to avoid patients coming into surgeries were recommended [1].

Prescribing is the most common intervention in healthcare and comprises the second highest spend in the NHS, after personnel costs.

**Fig. 1:**
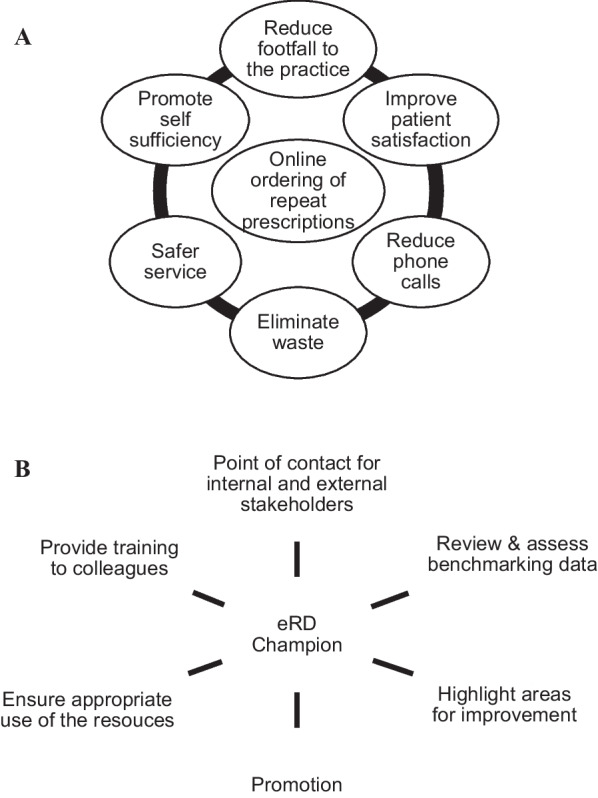
**A** Summary of the benefits of online prescription ordering in general practice, **B** the role of the eRD champion in a general practice setting (adapted from Wessex AHSN [7])

Repeat prescriptions form approximately 60–75% of all prescriptions written in primary care chronic and long-term conditions and account for approximately 80% of the costs [2]. Typically, these prescriptions are requested from the GP surgery by the patient in several ways, this includes online requests via designated portals or physically bringing in a written request to the GP surgery. Many surgeries across the UK have moved away from telephone requests for prescriptions in line with NHS England guidance [3] with the benefit of online ordering (Fig. 1A).

Repeat dispensing (RD) is part of the repeat prescribing process that allows the healthcare professional (HCP) to issue a number of batch prescriptions for a specified period of time and this can be via electronic repeat dispensing (eRD) or via paper prescriptions whereby once the batch of prescriptions is issued, in each instance the next supply of medication is needed, this is managed by the patient’s pharmacy of choice [4]. The large volume of repeat prescribing in general practice is a huge amount of work and therefore a robust and efficient repeat prescribing system is recommended to be implemented in general practice and that the system should be overseen and managed by an appropriately trained individual, with defined deputy and cover arrangements [5].

The initial pilot for RD was undertaken in 2005 whereby a service specification and regulations outlined how the service should be provided and the requirements for community pharmacies to have relevant and appropriate governance arrangements for the management of the service [6].

Wessex Academic Health Sciences Network (AHSN) have undertaken a considerable amount of work on eRD and concluded that there are significant savings in general practitioner (GP) time to be made from moving to eRD and in some instances this may be up to 45 min per day, per practice [7]. The estimate is that up to 80% of all repeat prescriptions may be suitable for eRD and could potentially save 2.7 million hours of GP and practice time [8]. From April 2019, eRD was included as part of the core GP contract for patients to be commenced on eRD where clinically appropriate with patient consents [9] and consequently the need for patient consent was temporarily removed in June 2020 [10]. eRD was the next addition to the electronic prescribing service (EPS). The purpose of the batch prescription was to eliminate the need for patients to order the same prescription monthly and for this to be processed monthly by the GP surgery staff and authorised by a prescribing clinician.

To understand the scale of repeat prescribing across the UK, a significant proportion of the population take medications for chronic diseases and are for long-term use. This increases with age with data showing that 77% of issued items were for repeat prescriptions with a mean of 3.9 items per patient per annum [11]. Despite eRD now forming part of the core GP contract, uptake rates have always remained low, with the mean uptake across Clinical Commissioning Groups (CCGs) across West Yorkshire practices (March 2020) being just above 16%, whereas nationally the uptake is as high as 75%. Repeat dispensing has been found to have clear benefits to patients, GP practices and pharmacies, however, several barriers were identified, such as understanding of the repeat dispensing process and time taken to implement [11].

The current COVID-19 pandemic has also placed huge strains on these repeat prescribing systems in general practice and has had consequent effects on community pharmacy workloads. The huge increase in workloads for community pharmacy has also been reported in the pharmacy press [12]. In addition, in line with the current position statement of NHS England and Wessex AHSN that an 80% uptake of eRD of all repeat prescribing can release GP and practice time of 2.7 million hours annually in England. This necessitates a greater adoption of eRD in general practice settings and consequent benefits to community pharmacy and ultimately patients.

### The eRD champion as the change agent

A published eRD handbook by Wessex AHSN was updated in May 2020 as part of the wider NHS England drive to improve the uptake of eRD across England. The recommendation that a key success criterion is that each surgery and community pharmacy must nominate one or more eRD champion(s) [7]. They define the eRD champion as ‘a member of staff who can promote the use of the scheme internally, aid the liaison with their practice and community pharmacy colleagues, and maintain momentum in the drive to increase the utilisation of RD via electronic means such as eRD’. Whilst the role of the champion is deemed an important success criterion, the aim is to ensure the whole wider interdisciplinary practice team understand eRD and that it becomes a part of routine practice.

The role of the eRD champion in general practice is summarised (Fig. 1B). There is significant literature around the role of change agent and in the context of eRD in general practice, this is most likely to be an internal change agent.

Change agency would be an appropriate way to describe the role of the eRD champion as stated in the Wessex AHSN eRD handbook. The change agent may lead the work as this individual will be tasked with initiating or facilitating a change programme. An internal change agent would be used by general practices and would typically be working in the surgery. Due to the governance requirements of NHS organisation’s personnel, it is unlikely that an external change agent would be able to fulfil this role with ease. It would be beneficial for the internal change agent to have sufficient authority and autonomy to be able to make decisions on the change process [13].

The roles which have independent prescribing have the authority and autonomy to be able to screen patients that may be suitable for and implement eRD during their routine work, whilst support the adoption of the workstream across the wider team and surgery.

In the case of improving eRD uptake, a social construction model of change agency aptly describes the change model needed. Social construction suggests that the change is based on a collaborative effort rather than being wholly dependent on one or two key individuals and the imperative is on the need to drive change through a method of co-construction that is not just restricted to the practice management team but involves personnel at all levels of the general practice [14].

Reviewing the uptake of eRD in the GP surgeries will enable us to understand the barriers to implementation. This will help to advise authorities in the NHS how to improve prescribing systems in general practice to streamline workflows and reduce the demand on the system during extreme workloads and workforce shortages using existing mechanisms such as eRD.

Therefore, the aim of the present study was to investigate eRD uptake in participating general practices across West Yorkshire. The objective was to understand the key enablers of eRD in this setting given eRD was designated a priority workstream by NHSE during the pandemic and appraise the current evidence for the stated efficiency gains for implementing eRD.

## Method

### Participating groups and sampling

The population defined to participate in this study is those working in a general practice setting as they will all have some involvement in the prescribing process and therefore are identified as relevant stakeholders of this process, of which eRD is one aspect. This study was a voluntary, cross-sectional survey. The questionnaire was circulated to members of staff working within general practice settings across West Yorkshire, by utilising practice manager forums and established clinical networks within the CCGs across West Yorkshire; Bradford, Huddersfield, Calderdale, Leeds, and Wakefield and explicitly stated that only one questionnaire response per general practice was required. Prior to questionnaire completion, each respondent has the opportunity to review background information about the study, its purpose, length of the questionnaire and information about the research team and data management.

**Fig. 2:**
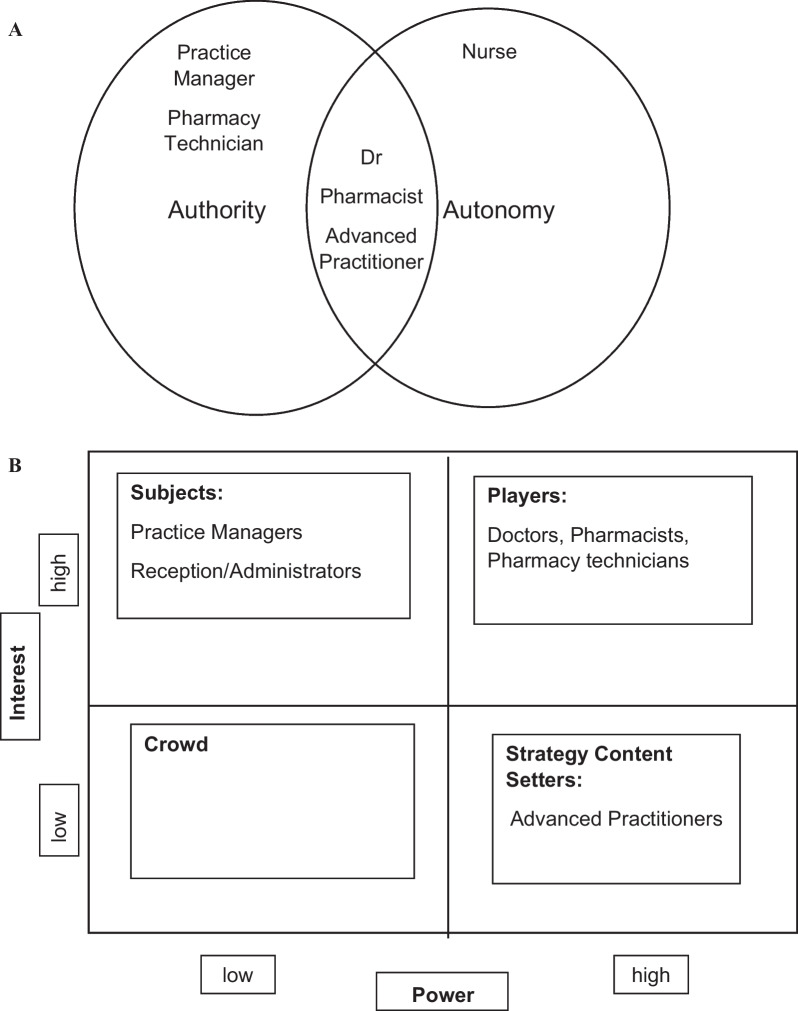
**A** Authority and autonomy of roles in general practice to implement eRD is linked to those HCPs with the right to prescribe independently (adapted from Jabri [13]). **B** eRD Stakeholder mapping according to the Interest vs. Power Matrix in General Practice (adapted from Eden and Ackermann, 1998)

A stakeholder analysis which forms the basis for their inclusion in this study and remains the only inclusion criteria, has been summarised (Table 1). By viewing each identified role within the general practice as stakeholders of the process in terms of their ability to influence the repeat prescribing process (Fig. 2A), it is additionally useful to understand and classify them in an Interest vs. Power Matrix (Fig. 2B). An analysis of this matrix suggested that pharmacists and pharmacy technicians represent the groups with the highest interest and power to implement eRD within the general practice, which is understandable given their role has a primary focus on prescribing. The doctor’s role within general practice is multifactorial and despite having high power may not have a high interest in the uptake of the service. The practice manager will have high interest in implementing the service, due to identified potential efficiencies, but will not be directly involved in undertaking the service itself. As all identified stakeholders are involved in the service, all were invited to participate in the study and based on self-selection sampling [15]. Four groups were identified as the targeted participants in this study, although, any role with an involvement in the repeat prescribing process would be able to participate. Questionnaire completion implied a consent to participate.

**Table 1:** Internal stakeholder analysis in general practice (adapted from source: Wessex AHSN [7])

Role	Involvement in eRD	Potential benefits from the increased uptake of eRD
Clinician (Dr, Nurse, Advanced Practitioners, Pharmacists, Pharmacy Technicians	Setting up eRD for appropriate patients after clinical review Lead eRD service delivery	Time capacity released by not having to undertake repetitive tasks on a monthly basis
Practice Managers	Receive benchmarking information for NHS Responsible for ensuring resources, systems, and Processes in place for service delivery Ensuring learning and development undertaken for identified roles Reviewing performance and monitoring of uptake	Increased process efficiency and capacity released in the clinical team to undertake other tasks
Reception/Administrators	Promote the service to the end users, i. e. patients Receive queries around the service itself as the first contact Can champion the service	Reduces the flow of end users, i. e. patients who need to contact the general practice with requests for medication

### Questionnaire

A questionnaire was devised based on existing policy documentation [4–7, 9] and in consultation with GP practice staff, cognitive interviews, and the ethical committee at the University of Huddersfield. This consultation identified three themes to focus on and included: (a) benchmarking, (b) learning and growth, and (c) internal processes. The questionnaires were sent to potential participants between July 2020 and November 2020.

This generated data for quantitative analysis with the data collected through the survey method used to suggest the possible relationships between variables and allowed the models of these relationships to be developed as appropriate and, importantly if the relationship between the variables is significant, produce models of these relationships.

Regarding the time horizon of the study, a cross-sectional approach was adopted.

### Data analysis

Quantitative analysis was undertaken to the responses of the questionnaire using SPSS (V25, International Business Machines Corporation (IBM), Armonk, New York, USA) for statistical analysis. Descriptive statistics were expressed as the number of respondents (% of total). The association between continuous variables was determined using Pearson correlation coefficient based on the method of covariance. Statistical significance was set at *P* < 0.05.

### Ethical considerations

The University of Huddersfield granted ethics approval for this research, ethics approval number SAS-SREIC 17.06.20-1A.

## Results

### Participant demographics and benchmarking

**Fig. 3:**
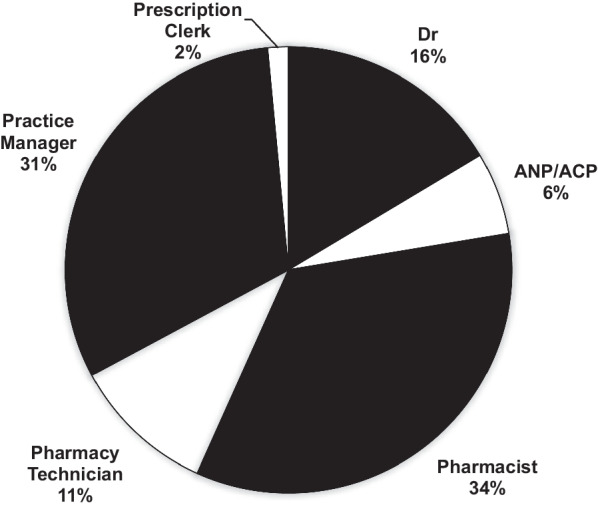
Pie chart showing the job roles of the participants

A total of 67 responses collected from both male and females at five different age ranges starting from 26 to over 65 years old working at different practices. The age range of 26–35 was the highest represented and female participants representing 66% of the total participants. The job roles of participants are reported in Fig. 3.

Only 40 participants were aware of the uptake of eRD (59.09%). Of the 59.09% of participants that were aware of their eRD uptake, they provided further detail on the actual % of the uptake, and 27 participants (41.91%) were not aware of eRD (Table 2).

**Table 2:** Participant awareness of uptake of eRD

	Frequency	Percent	Valid percent
*Valid*			
0–10%	16.0	23.9	40.0
11–20%	10.0	14.9	25.0
21–30%	7.0	10.4	17.5
31–40%	3.0	4.5	7.5
41–50%	2.0	3.0	5.0
51–60%	2.0	3.0	5.0
Total	40.0	59.7	100.0
Missing system	27.0	40.3	
Total	67.0	100.0	

The mean value for the uptake of eRD was 4.56% ± 0.229%. The mode was between 0 and 10% and median was between 0 and 10% which had the highest frequency and the frequency decreased as the percentage uptake increased to 11–20%.

### Use of learning resources

**Fig. 4:**
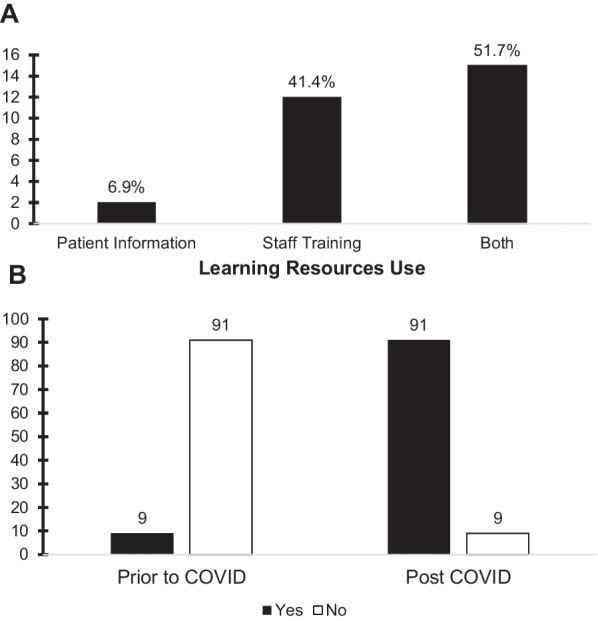
**A** Histogram showing in those participants where NHS learning resources had been used and the use case of the resources.** B** Histogram showing % of which general practices were taking prescription requests over the prior to COVID-19 and post COVID-19 lockdown measures in March 2020

Learning resources had been utilised by 29 participants who had used the NHS learning resources for eRD (43.3%) and 38 participants did not use the resources (56.7%). Further information requested on the use of e-learning resources indicated they were being used for: (1) patient information, (2) staff training, and/or, (3) both. Results showed that only 6.90% of the participants used resources to aid and supplement patient understanding, 41.38% used the resources for staff training and 51.72% of participants took part in both activities (Fig. 4A).

### Prescribing related workload

9% of general practices received prescription requests on the telephone prior to COVID-19 pandemic and this was significantly increased to 91% during the pandemic in March 2020. Furthermore, 37.31% of the practices had a prescription line to take prescription requests over the phone during the pandemic, however, majority of the practices (62.69%) did not (Fig. 4B).

### Repeat prescription authorisation and service lead

When participants were questioned about the involvement in repeat prescription authorisation, results showed that pharmacists were very much involved (82.1%) as compared to GPs (79.1%), advanced nurse practitioners (ANP)/advanced clinical practitioners (ACP) (52.2%), pharmacy technicians (19.4%), other staff at GP practices (14.9%) and nurses (7.5%). The authorisation period, after a medication review had been undertaken, varied from 3 to 12 months.

**Fig. 5:**
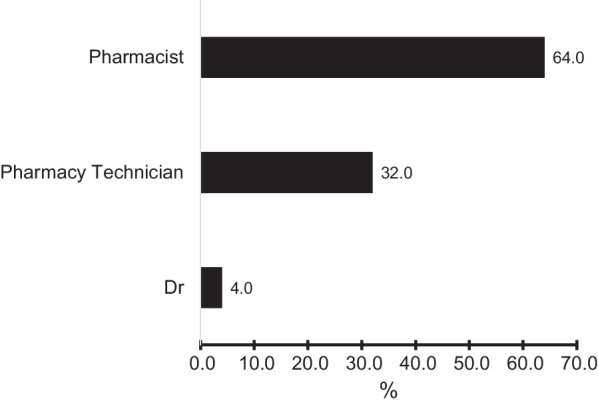
Histogram showing which HCP was nominated as eRD service lead

The number of practices that integrated eRD in their workflow was only 34.33%. In addition, only 37.3% of the practices had an eRD service lead nominated which were mainly pharmacists (64%), pharmacy technicians (32%), and GPs (4%) (Fig. 5).

Integrating eRD into normal workflows regarding the repeat prescription authorisation process (Pearson correlation coefficient, *P* < 0.001) and having a nominated eRD champion in the practice (Pearson correlation coefficient, *P* = 0.04) resulted in higher uptakes of eRD in the participant practices.

## Discussion

### Benchmarking

With the increasing availability of data generated within healthcare systems, there is a need to continually identify reliable methods for the mapping and measuring quality of care [16]. Benchmarking in the context of healthcare can be described as the ‘continuous, systematic search for, and implementation of, best practices that lead to superior performance’ [17]. NHS England comparative benchmarking data for eRD are widely available to general practices and often communicated with each general practice by their local CCG. The average uptake across West Yorkshire is 16% [18]. In contrast, the uptake of participant practices is below the average uptake for West Yorkshire. More recently, benchmarking data have been misused in healthcare settings and has become more associated with performance management rather than what it is intended for, i.e. contributing to best practice when delivering healthcare services and the management of organisations [17].

With more participants having accessed their data, 59.09%, it shows good awareness of eRD, however, more work can be done to improve this among the other participants’ practices. This study shows that a majority of participants used benchmarking data; this contrasts with some studies that suggest that benchmarking data were generally underused by healthcare decision-makers [19, 20], and there may be a reluctance to integrate benchmarking data to change behaviour and procedures. The degree of benchmarking information integration varies between clinicians and healthcare managers [21] and highlights the importance of undertaking an exercise as described in the interest versus power matrix (Fig. 2B). NHSE makes it easy to access these comparative benchmarking data making them widely available to general practices and often communicated with each general practice by their local CCG.

### Learning resources

NHS England has provided specific learning resources for personnel in general practice for upskilling with respect to the eRD service and are freely available. Results showed that 43% of the participants had used the recommended eRD resources. Of the participants that used the NHS learning resources, 52% of the respondents were using the learning resources for both staff training and for patients and 41% using them for staff training alone. This type of learning by NHS personnel has been described as single loop (or adaptive) learning by the individual and aims to make changes to working procedures [22]. With the learning resources used by both staff and patients, this was perhaps a missed opportunity for the participant practices to undertake double loop (or generative) learning in conjunction with patients to embed the service, although the opportunities for patient interaction may have been impacted by the COVID-19 restrictions in place. This approach is encouraging as it demonstrates that general practices have identified both groups as important stakeholders in the eRD process and are taking actions to address the needs of both groups. As many patient resources focus on effective communication and understanding of the eRD service, this may have been a missed opportunity for these participant’s surgeries. The role of community pharmacist in patient education and information has been highlighted during the COVID-19 pandemic [23] and this may have additional opportunities for the use of resources by patients and support community pharmacies with increased workload and dispensing demands.

### Prescribing related workload

The increase in accommodating telephone requests for repeat prescriptions is in keeping with the total triage system of contact for patients during the COVID-19 pandemic and for contact to be mainly via non-face to face methods. As the number of practices that are taking prescription requests over the phone, 37.71% have a dedicated prescription line or time slot for this with the remainder allowing these requests to happen any time during the opening hours of the surgery. Most of the participants described the workload associated with taking prescription requests over the telephone as busy or very busy.

The Kings Fund highlighted that there is no routine monitoring activity undertaken by NHS bodies on GP activity data and only secondary data are available via the GP clinical system providers and their respective research arms [24]. Their research and analysis indicated that between 2007 and 2014, there was a 10.5% increase in GP and nurse consultations in general practice, alongside an increase in average length of each consultation and direct patient facing clinical workload. This did not consider the indirect activities and other professional duties that are essential and suggested that English primary care in its current state may reach saturation point soon [24, 25]. These workload challenges have only been amplified during the COVID-19 pandemic and have placed challenges on general practice teams and the data support this with most participants, combined 89.55%, describing their prescription related workload as busy or very busy.

### Repeat prescription authorisation

Several HCPs are involved in this process and increasingly pharmacists are leading this workstream, in line with the development of primary care as per the NHS Long Term Plan to build resilience, increase capacity, promote interdisciplinary working, and improve patient outcomes [26]. Seeing this trend of prescribing activities changing from a Dr to other HCPs and in particular pharmacists is encouraging and ensures some resilience and increased efficiency in the prescribing system with an expected capacity release for doctors becoming available to be utilised elsewhere. A report from the Royal College of Physicians highlighted the extent of the shortage of doctors in the UK with the causes being multifactorial [27], therefore being able to release capacity in general practice with the already existing provision can only be of benefit to the general practice itself and for patient care and outcomes, with this capacity release being enable by increased utilisation of eRD.

Prescription authorisation is a crucial stage of the prescribing process for those patients on long-term medication and would be the most relevant point at which to assess suitability and implementation of eRD.

Most of the participant surgeries opted for a 6- or 12-month period as the main options of repeat prescription authorisation and is often a balance of patient stability and safety.

Numerous sources have identified that the aim should be to embed eRD into routine prescribing workflows in the general practice [7, 9]. Having eRD as a routine part of prescribing workflows would come further downstream as an objective and would fall under the premise of sustaining change. If a practice had a low uptake as there would be other tasks that would need to be completed first to ensure readiness for change and implementing the service. May et al., advocated that planned improvements are embedded in routine practices and integrated into the normal organisational processes to prevent relapse [28]. They described this as ‘normalisation’. Normalisation is crucial to achieve, as when the planned activity is no longer supported by a specific project or team, the chances of relapse are high.

### Service lead

Having an eRD champion is listed as one of the key criteria for success in increasing the implementation of the service. The eRD champion would fulfil the role of the change agent and the evidence behind the use of change agent is substantive [13, 29]. In all cases as part of the study, this change agency came from within the general practice, i.e. internal change agent and the leading roles of the change agent were either pharmacist or pharmacy technician.

With the two roles becoming more ubiquitous in general practice settings since the NHS Long Term Plan was announced with dedicated funding for pharmacists and pharmacy technicians [26] with the focus of these roles supporting the whole prescribing process, it is these individuals who will most likely have volunteered or have been allocated the role of eRD champion. This study did not investigate whether the service leads were volunteered or allocated.

The pharmacist role has sufficient autonomy and authority to lead the implementations of eRD whereas it could be considered that the role of the change agent may be less implementing the eRD themselves and act in the capacity as the champion and ensure implementation by collaborative working with others, who have the autonomy to implement eRD, and fulfil the co-constructionist model of change agency to a higher degree than an individual working alone who may have authority and autonomy. Both roles prominently featured as having both power and interest in eRD.

Higher uptake of eRD was demonstrated where the general practice integrated eRD into routine workflows during the repeat prescription reauthorisation process and where an eRD service lead is nominated. The time when a clinical review is being carried out and the HCP will authorise the medication to be taken for a defined period, after a medication review, and as such it is an opportune time to initiate eRD for the patient. As part of the clinical review happening at this point, it is likely to involve some patients contact and therefore it additionally lessens the need to make an additional contact with the patient at a separate time to commence eRD. The presence of an eRD champion will make this more likely to happen and contribute to an increased eRD uptake. In a systematic review on the sustainability of pharmacist services has shown that the presence of a leader or champion to guide and support the service and its adaptation may support the service becoming routine practice and that it may continue to provide benefits once the implementation phase is completed [30].

### The economics and efficiencies of repeat prescribing

The prescribing of medicines has seen a year-on-year increase and in 2015, 1.08 billion prescription items were issued by general practices in England, an average of 200 prescription items per GP per week, and this represented a 1.8% increase on the previous year [31]. The total net ingredient cost (NIC) for prescription items dispensed in 2017 was £9.17 billion [32]. As 80% of all prescriptions issued are for long-term repeat prescriptions, the cost of repeat prescribing in England in 2017 is approximately £7.34 billion.

NHS Digital advises that 80% of repeat prescription medicines can be ordered by repeat dispensing and using RD by electronic means, that is, eRD. Therefore, on the data available from NHS England, eRD has a maximum uptake of 864 million prescription items in England and could save 2.7 million hours of GP and practice time annually [33].

In April 2000, the Prime Minister announced several areas that, if developed and implemented, may lead to a faster and more efficient service from GPs to patients, and provision of repeat prescriptions was highlighted as part of this report [34]. To understand the burdens of GPs, the analysis involved a series of face-to-face interviews with doctors in general practice. This report predates the roll out of electronic transmission of prescriptions (ETP) and highlighted that at the time, there were 410 million repeat prescriptions generated every year which was the equivalent to an average of 200 GP hours per week. At the time, this represented a major burden on general practice and GP time across the country. The suggested course of action identified by the Department of Health was that the greater utilisation of repeat dispensing up to a rate of 80% of all repeat prescriptions may potentially yield a saving of up to 2.7 million hours of GP and their practice time and increase patient satisfaction.

The estimates of potential time savings are the lower of the 95% tolerance limit on the median number of requests for repeat medications that were made within the data collected. The amount of time consumed by these requests has been calculated from the product of the mean time value reported to undertake the requests for repeat prescriptions and the lower tolerance interval for number of requests per GP used to calculate the potential time saving. The report provides no further details as to the number of responses received and the quantification process of the potential savings of 2.7 million hours of GP and practice time savings.

## Conclusion

This study was carried out to understand the enabling factors that can lead to the implementation of eRD in general practices. Two main enabling factors were identified within this study that may lead to improved implementation and uptake of eRD, integrating eRD into normal routine workflows where prescribing authorisation is concerned and nominating an internal eRD champion to really lead and drive the service internally, although, service adoption may rely on balancing fidelity with adaptability. Data used to inform current policy on efficiency gains do not reflect current prescribing processes and further research and validation is needed to support policy development.

## Recommendations

For general practices to consider utilising eRD further in their respective practices, the following should be considered:

1. Appointment of an eRD champion in the practice. This individual should be familiar with the dynamics of the organisation and with sufficient authority, autonomy, and interest in prescribing. Roles that may be suitable for an eRD champion are a pharmacist and pharmacy technician.
2. In alignment with the NHS Long Term Plan, additional capacity and funding is available to recruit pharmacists and pharmacy technicians to work in a locality of general practices, now termed Primary Care Networks. These individuals may be suitable for the role of eRD champion with the added benefit of working across sites in a locality, this individual may be critical to increasing the uptake of eRD in more than one general practice at a time and be able to share good practice from one site to another.
3. Integrating and embedding eRD into routine workflows for prescribing is essential to ensure that eRD uptake is implemented and allows for the service to remain sustainable. This ensures the general practice can continue to benefit from increased efficiency and released capacity.
4. The learning undertaken for staff and the messaging for patients and signposting to NHS resources could be discussed with the patient participation groups and the general practice staff. This will help foster a dialogic climate between important stakeholder groups and aid in co-learning within the surgery.

## Limitations and further study

The results of one case research activity cannot be generalised. Despite the questionnaire revealing valuable insights into what the enabling factors may be, the small sample size limits the explanatory power of quantitative analysis. Further study to increase the sample size and include more general practices over a larger geography and longer time frame alongside an analysis into the characteristics needed to be an effective eRD champion is warranted. Further research is required to validate the stated efficiency gains that may be achieved in general practice. This can be done by increasing the use of eRD, as an NHS England priority workstream. This is important as the current position statements are generated from research that predates the implementation of ETP and fails to acknowledge the impact ETP may have had on repeat prescribing systems.

## Data Availability

The datasets used and/or analysed during the current study are available from the corresponding author on reasonable request.

## Abbreviations

ACP: Advanced clinical practitioners
AHSN: Academic Health Sciences Network
ANP: Advanced nurse practitioners
CCG: Clinical commissioning groups
EPS: Electronic prescribing service
eRD: Electronic repeat dispensing
GP: General practitioner
HCP: Healthcare professional
RD: Repeat dispensing
